# Efficacy and Safety of a New Botulinum Toxin Type A Free of Complexing Proteins

**DOI:** 10.3390/toxins8010004

**Published:** 2015-12-24

**Authors:** Hyun-Mi Oh, Joo Hyun Park, Dae Heon Song, Myung Eun Chung

**Affiliations:** 1 Department of Rehabilitation Medicine, Seoul St. Mary’s Hospital, College of Medicine, The Catholic University of Korea, 222 Banpo-daero, Seocho-gu, Seoul 06591, Korea; hazel@cmcnu.or.kr (H.-M.O.); drpjh@catholic.ac.kr (J.H.P.); 2 Department of Rehabilitation Medicine, St. Paul’s Hospital, College of Medicine, The Catholic University of Korea, Wangsan-ro 180, Dongdaemoon-Gu, Seoul 02559, Korea

**Keywords:** botulinumtoxins, type A, efficacy, safety, clinical trial

## Abstract

MT10107 is botulinum neurotoxin type A derived drug which utilizes the 150 kDa portion without complexing proteins and human serum albumin contents. To evaluate the efficacy and the safety of MT10107, it was compared with onabotulinumtoxinA in this double-blind, randomized controlled trial. Twenty-five healthy males received a randomly selected dose of MT10107 into the extensor digitorum brevis (EDB) muscle of one foot, and an equivalent dose of onabotulinumtoxinA (BOTOX) was injected into the contralateral EDB muscle. While efficacy of the administered substance was determined by measuring paretic effects on the EDB, the local spread of toxin effects was evaluated by the paretic effects on the nearby abductor hallucis (AH) and abductor digiti quinti (ADQ) muscles. Paretic effects were defined as the percentage of reduction of the compound muscle action potential (CMAP) amplitudes, measured at 14, 30, 90 days after the injection, compared to the baseline value. Intergroup (MT10107 and onabotulinumtoxinA) differences were not significant in the percentage reduction of the amplitudes in the EDB muscles. In this study, there was no significant difference in efficacy and safety between the two test drugs. MT10107 may be effective and safe as much as onabotulinumtoxinA to produce the desired paretic effect.

## 1. Introduction

In past decades, botulinum neurotoxin type A (BoNT/A) has been used as treatment for neuromuscular disorders such as blepharospasm, strabismus, hemifacial spasm, and cervical dystonia. In addition, it has been applied in aesthetic facial rejuvenation including effacement of rhytides by inhibiting the contraction of facial expression muscles [1].

BoNT/A, the most potent of all the subtypes, contains complexing proteins (CPs) that include one toxic protein and one or more nontoxic proteins like such as hemagglutinins or nontoxic nonhemagglutininproteins. Among the preparations of BoNT/A on the market, incobotulinumtoxinA (Xeomin, Merz Pharmaceuticals, Frankfurt am Main, Germany) is the only complexing protein-free BoNT/A [2]. In several established studies, incobotulinumtoxinA has presented similar efficacy and safety, compared with the onabotulinumtoxinA (BOTOX, Allergan, CA, USA) which contains nontoxic proteins [2,3,4,5]. However, some studies suggest that the potency of incobotulinumtoxinA is inferior to that of onabotulinumtoxinA or incobotulinumtoxinA due to a shorter treatment duration than onabotulinumtoxinA [6,7]. For example, in the study of Moers-Carpi *et al.*, 20 units of onabotulinumtoxinA were as effective as 30 units of incobotulinumtocinA. This study supports the non-interchangeability of unit doses of BoNT/A [8].

Like the incobotulinumtoxinA, MT10107 (Meditox, Chungcheongbuk-do, Korea), including the toxic part (150 kDa), is a highly purified BoNT/A preparation without any aforementioned nontoxic molecular protein. MT10107 is free of human serum albumin which acts to stabilize the neurotoxin complex and to prevent it from aggregation on surfaces. In MT10107, methionine, polysorbate 20 and sucrose are used as stabilizers instead of human serum albumin. It is in a freeze-dried format; it can be used after diluted with normal saline. To evaluate the biological potency of BoNT/A, LD50 assay was performed according to the European pharmacapoiea. The results were analyzed with a quantal response parallel-line probit. When a rat was injected with MT10109, the range of the lethal dose was between 50 units/kg and 200 units/kg for both sexes [5].

There are preliminary animal studies that suggest the possibilities that complexing proteins in BoNT/A might cause the formation of neutralizing antibodies against BoNT/A, which could possibly lead to treatment failure. Frevert and Dressler suggest that incobulinumtoxinA presented a lower risk of generating antibodies. In addition, other preliminary experiments with incobulitunumtoxinA suggested that absence of the complexing protein was associated with reduced immunogenicity [9]. The purpose of this present study was to determine the efficacy and safety of MT10107 by comparing it with onabotulinumtoxinA, the well-known BoNT/A preparation, in the target muscle, the extensor digitorum brevis (EDB), of healthy male volunteers.

## 2. Results

### 2.1. Baseline Demographic

Enrolled participant characteristics are presented in Table 1. All the enrolled volunteers were healthy male subjects who were in their twenties 20s and thirties 30s except for one 50-year-old subject. There was no significant difference for age, body weight, height, and preferred foot among the five dose-response groups (*p* = 0.710, 0.420, 0.566, 0.167, respectively).

**Table 1:** Baseline characteristics of 25 participants according to the randomly assigned group.

Characteristic	2U Group (*n* = 5)	5U Group (*n* = 5)	10U Group (*n* = 5)	20U Group (*n* = 5)	30U Group (*n* = 5)	*p-*Value
Gender Male	100%	100%	100%	100%	100%	-
Age (years) Median	26	31	31	28	27	0.7101 *
Range	23–33	21–34	21–50	25–36	25–30	-
Body weight (kg)	74.30 ± 6.79	73.24 ± 9.30	78.22 ± 7.80	80.92 ± 12.93	71.22 ± 4.04	0.420 *
Height (cm)	179.74 ± 5.79	174.72 ± 8.02	175.40 ± 3.74	174.14 ± 3.46	175.04 ± 6.56	0.566 *
Right preferred foot	100% (5)	100% (5)	100% (5)	100% (5)	60% (3)	0.167 ^†^

Plus-minus values are means ± SD; * *p* values are based on ANOVA test; ^†^ *p-*values are based on Fisher’s exact test.

### 2.2. Efficacy

**Figure 1:**
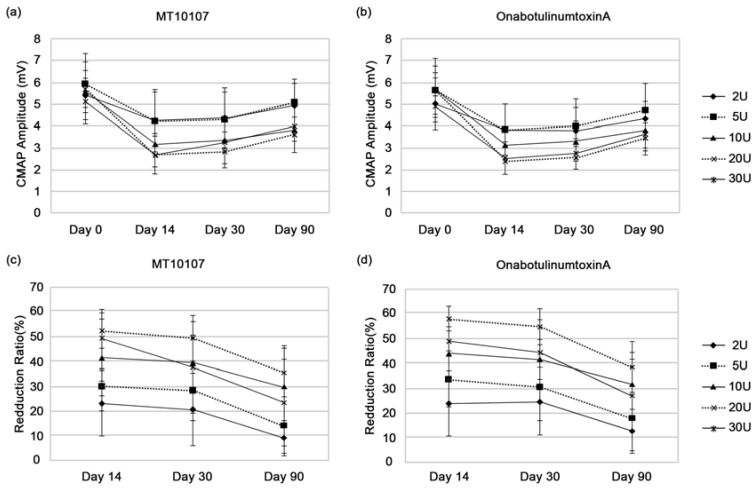
Comparative assessment of the two different toxins over time, following injection of paired MT10107 and onabotulinumtoxinA, one injected on the right extensor digitorum brevis (EDB) muscle and the other on the left one. Dose response of the compound muscle action potential (CMAP) M-wave amplitude with the two different toxins at the EDB site (**a**,**b**). Changes in the percentage of reduction of the EDB CMAP M-wave amplitude compared to the baseline in each dose group, at 14, 30, and 90 days (**c**,**d**). Each point is the mean ± SD, *n* = 5.

Efficacy was determined by measuring the paretic effects on the EDB muscle after the injection by calculating percentage change. After MT10107 and onabotulinumtoxinA injections, the percentage reductions of the EDB compound muscle action potential (CMAP) M-wave amplitudes compared to the baseline values were statistically significant for the study and the control groups at each time point (post-injection days 14, 30, and 90) and among all dose groups (Figure 1). Differences in the percentage reduction of CMAP M-wave amplitude measured in EDB after injection of paired MT10107 and onabotulinumtoxinA manifested no significant difference between the study and the control groups in any dose groups and at any time point (Table 2).

**Table 2:** Differences in the percentage reduction (%) of CMAP M-wave amplitudes measured in EDB after bilateral injections of paired MT10107 and onabotulinumtoxinA.

Difference ^a^	Day 14		Day 30		Day 90	
Dose Group	Difference ^a^	*p*	Difference ^a^	*p*	Difference ^a^	*p*
2U	−0.75 ± 2.49	0.5376	−3.81 ± 3.17	0.0550	−3.42 ± 3.32	0.0825
5U	−3.44 ± 4.12	0.1354	−2.15 ± 5.12	0.4002	−3.61 ± 12.14	0.5430
10U	−2.10 ± 4.47	0.3527	−2.30 ± 3.58	0.2230	−1.82 ± 6.56	0.5685
20U	−5.49 ± 8.78	0.2344	−5.31 ± 10.03	0.3016	−2.89 ± 7.25	0.4228
30U	0.31 ± 8.21	0.9363	−6.38 ± 16.93	0.4467	−3.50 ± 14.94	0.6277

Mean values (%) ± SD; *p*-values are based on paired *t*-test; EDB, extensor digitorum brevis; CMAP, compound muscle action potential; ^a^ Difference between percentage reduction of CMAP M-wave amplitude measured in EDB after injection of MT10107 and that after injection of onabotulinumtoxinA.

### 2.3. Spread of Toxin Effects

To investigate the local spread effects of MT10107, the percentage changes in the CMAP M-wave amplitudes of the adjacent muscles, the abductor hallucis (AH) and abductor digiti quinti (ADQ), were measured in all of the participants in all dose groups at each return visit and compared to e baseline values. For the AH muscles, the changes compared to the baseline values in the 20U dose group were significantly greater (*p* = 0.0251) at 90 days after injection. Except for this one instance, there was no significant change at any time point, either in the study or the control groups, at any dosage (Table 3). For ADQ muscles, the other adjacent muscle, the changes compared to the baseline were significantly inferior in the 5U dose group at Day 14 and in the 2U dose group at Day 90 (*p* = 0.0474 and *p* = 0.0371, respectively), whereas no significant reduction was exhibited at any other time point in any other dose groups (Table 4). There was no significant difference in the local spread of toxin effects of the two preparations on both adjacent muscles.

**Table 3:** Mean percentage reduction (%) of AH CMAP M-wave amplitudes compared to baseline after MT10107 and onabotulinumtoxinA injection.

Reduction	Day 14		Day 30		Day 90	
	Reduction	*p*	Reduction	*p*	Reduction	*p*
**MT10107**						
2U	0.38 ± 0.34	0.2500 ^b^	0.32 ± 1.57	0.6694 ^a^	−1.21 ± 1.55	0.1560 ^a^
5U	0.33 ± 0.53	0.5000 ^b^	−0.02 ± 0.59	0.9518 ^a^	0.08 ± 3.78	0.9625 ^a^
10U	−0.11 ± 0.28	0.4326 ^a^	0.08 ± 0.76	0.8266 ^a^	−0.01 ± 1.29	0.9829 ^a^
20U	−0.23 ± 0.38	0.5000 ^b^	−0.67 ± 1.09	0.2500 ^b^	−3.17 ± 2.03	0.0251 ^a^
30U	0.24 ± 0.52	0.3579 ^a^	1.24 ± 3.09	0.8750 ^b^	1.11 ± 4.09	0.5763 ^a^
**OnabotulinumtoxinA**						
2U	−0.28 ± 0.45	0.5000 ^b^	−0.32 ± 1.31	0.6118 ^a^	3.27 ± 7.09	0.3606 ^a^
5U	0.20 ± 2.32	0.8591 ^a^	0.29 ± 3.61	0.8675 ^a^	−0.33 ± 2.27	0.7626 ^a^
10U	4.20 ± 8.27	0.2500 ^b^	2.14 ± 4.35	0.3125 ^b^	1.62 ± 4.60	0.6250 ^b^
20U	−0.03 ± 0.70	0.6250 ^b^	0.34 ± 1.32	0.6019 ^a^	−1.79 ± 2.65	0.2064 ^a^
30U	2.03 ± 4.18	0.2500 ^b^	2.7 ± 3.71	0.0625 ^b^	1.17 ± 2.28	0.3167 ^a^

Mean values (%) ± SD; ^a^ *p*-values are based on paired *t*-test; ^b^ *p*-values are based on Wilcoxon sign rank test; CMAP, compound muscle action potential.

**Table 4:** Mean percentage reduction (%) of ADQ CMAP M-wave amplitudes compared to baseline after MT10107 and onabotulinumtoxinA injections.

Reduction	Day 14		Day 30		Day 90	
	Reduction	*p*	Reduction	*p*	Reduction	*p*
**MT10107**						
2U	0.00 ± 0.00	0.9028 ^a^	1.71 ± 3.23	0.3010 ^a^	1.62 ± 1.18	0.0371 ^a^
5U	0.81 ± 0.64	0.0474 ^a^	0.89 ± 2.17	0.4088 ^a^	−1.28 ± 4.73	0.5778 ^a^
10U	0.64 ± 3.02	0.8750 ^b^	1.15 ± 3.52	0.5045 ^a^	−0.45 ± 4.06	0.8146 ^a^
20U	0.69 ± 1.05	0.5000 ^b^	2.04 ± 3.06	0.1250 ^b^	−0.23 ± 0.79	0.5469 ^a^
30U	2.22 ± 6.38	0.8125 ^b^	1.46 ± 7.27	0.6762 ^a^	3.24 ± 7.29	0.3765 ^a^
**OnabotulinumtoxinA**						
2U	−0.62 ± 0.79	0.1545 ^a^	−1.12 ± 1.32	0.1299 ^a^	0.15 ± 3.82	0.9351 ^a^
5U	0.34 ± 0.75	0.3678 ^a^	0.92 ± 2.36	0.7500 ^b^	−0.45 ± 4.28	0.8240 ^a^
10U	1.02 ± 2.82	1.0000 ^b^	1.76 ± 2.67	0.2500 ^b^	−0.87 ± 2.30	0.4444 ^a^
20U	−0.41 ± 1.19	1.0000 ^b^	−0.22 ± 1.17	0.6946 ^a^	−1.20 ± 1.83	0.2174 ^a^
30U	0.88 ± 1.42	0.5000 ^b^	0.71 ± 1.88	0.4441 ^a^	1.55 ± 1.94	0.1468 ^a^

Mean values (%) ± SD; ^a^ *p*-values are based on paired *t*-test; ^b^ *p*-values are based on Wilcoxon sign rank test; CMAP, compound muscle action potential; ADQ, abductor digiti quinti.

### 2.4. Safety

During the study period, there were a few reports on drug-related local side effects in three out of the 25 subjects, including one case of pressure pain in the injected area and two cases of edema. All these subjects complained of mild symptoms on the injection sites of both feet; however, the symptoms disappeared within a few days.

There were a total of four subjects with general complications. There were one case of headache in the 2U dose group and one case of asymptomatic bacteriuria in the 5U dose group. In the 10U dose group, asthenia and T-wave inversion on the electrocardiogram were noted, respectively. The incidence of the complications was not significantly different among the all dose groups. There were no abnormal findings in laboratory tests, vital signs, and physical and neurological examinations after injections in this clinical study. Antibodies were not detected in any subject before or after the injection.

## 3. Discussion

In this present study, both the efficacy and the safety of MT10107 were assessed after injections into the EDB muscle of one foot and compared with onabotulinumtoxinA after the injection on the contralateral foot in healthy male adult volunteers. Such comparison methods have been widely utilized in previous studies that have measured the effects of BoNT. The validity of the procedures has been well established [4,10].

The paretic effects of the drug on the injected muscle indicate its efficacy. In this study, the percentage reductions of the CMAP M-wave amplitudes, compared to the baseline values, were measured via electrophysiologic methods using surface electrodes. Significant paretic effects were manifested in all of the various dosage groups of MT10107 and onabotulinumtoxinA. There was no significant difference in paretic effects between the bilateral sides of the EDB injected with preparation at any time point in all the dose groups.

According to our results, in the 30U dose groups, the mean percentage reductions of the CMAP M-wave amplitudes, compared to the baseline values, were found to be smaller than those in the 20U dose groups for both preparations at any time point, and in the 20U and 30U groups, even with the increased dose, the effects did not increase any more. This outcome might imply that the drug efficacy of the 30U dose is inferior to that of the 20U. Therefore, the equivalence tests were performed between the 20U and 30U dose groups to confirm their significance. On these multiple comparison tests, there was no significant difference between the two dose groups in the mean percentage reduction of the CMAP M-wave amplitudes compared to the baseline (Figure 1). This shows the similarity to those results in previously published studies presenting a logarithmic dose-response relationship between increasing doses and a decrease in EDB CAMP M-wave amplitudes [11]. This also might be due to the plateau effects that, at a certain point, even with an increased dose the effects did not increase. The small number of participants in each group (*n* = 5) might be another reason for such unexpected results, and we thought that a few patients in the 30U dose group might present non-satisfying responses to both preparations of MT10107 and onabotulinumtoxinA although the antibody to BoNT was not detected in any participants. Absence of detectable BoNT antibody may occur in patients with a poor response to BoNT injection [12].

To evaluate the local spread of toxin effects of MT10107, the percentage reduction of the CMAP M-wave amplitudes of the adjacent muscles, AH and ADQ, was measured after the drug injection and compared to the baseline values. For the AH, the percentage change compared to the baseline was −3.17% ± 2.03% in the MT10107 20U dose group at 90 days. For the ADQ, the change was 0.81% ± 0.64% in the MT10107 5U dose group at 14 days and also in the MT10107 2U dose group at 90 days; these data indicated significant differences. However, the changes were less than 20%, so it was difficult to interpret it as a clinical paretic effect. In addition, when both feet were compared, one injected for the study and one for the control, there was no significant difference in the changes of the adjacent muscle CMAP M-wave amplitudes compared to the baseline either between the two drugs or among all various dose groups at any point. These data implied that local spread of toxin effects of MT10107 or of onabotulinumtoxinA scarcely occurred in the dose range of 2U–30U [13].

Three subjects presented with local lower extremity side effects, including edema and pressure pain around the injection area. Without any treatment, they recovered within a few days without after-effects. During the study period of 90 days, there were no systemic adverse effects that necessitated an early termination of the study either with MT10107 or with onabotulinumtoxinA. A T-wave inversion, found on the electrocardiogram at 30 days after the injection, was not detected in the final electrocardiogram 90 days after the injection.

In this trial, amplitudes of the CMAP M-waves of the adjacent muscles, AH and ADQ, were measured in all of the participants in all dose groups. It was intended to present the effects of the diffusion of MT10107 compared with onabotulinumtoxinA. However, it was difficult to differentiate diffusion and local spread of toxin. This can be a limitation of this trial.

## 4. Experimental Section

### 4.1. Subjects

Twenty-five healthy male volunteers, 20–65 years old, were enrolled at a single center (St. Paul’s Hospital, Seoul, Korea). The demographic data about age, height and weight were recorded.

The baseline nerve conduction criteria for inclusion in this study were the following: CMAP M-wave amplitude of the EDB muscle was greater than or equal to 4.0 mV (average of the two largest CMAP amplitude values among the three measurements at baseline), and the CMAP M-wave amplitude of the AH and the ADQ muscles were greater than or equal to 5.0 mV. Exclusion criteria were any previous BoNT treatment during the past three months, history of childhood botulism, presence of a device implanted in the heart, and history of previous myotomy, denervation surgery or crush injuries in any of tested muscles. In addition, patients were excluded if they presented with an abnormality in serum or urine chemistry, a history of alcohol abuse, the regular administration of medications, or an allergic reaction to the agent used in this study. All volunteers had normal muscle power on the Medical Research Council scale, and there was no subject who ever received any form of BoNT before this study.

All participants gave informed written consent that they would be treated with an unlicensed and untested compound which might be potentially harmful. This study was approved by the Institutional Review Board of St. Paul’s Hospital (approval number: PC12BDSF006), and the Korean Ministry of Food and Drug Safety. This trial is also registered with the Clinical Research Information Service (Trial Number: KCT0000541).

### 4.2. Study Design

This was a randomized (by dose and injection side of foot), double-blind, dose-ranging, intra-individual, and single-center study done in 90 days. The 100U of MT10107 was compared with the 50U of onabotulinumtoxinA, the control agent. Both agents were prepared in five different dose groups: 2, 5, 10, 20, and 30U (Table 5). All solutions were contained in glass vials with concealed identifying labels for the blinded tests, and all injections were done with 0.1 mL of either agent.

**Table 5:** Dilution and injection volumes for paired MT10107 and onabotulinumtoxinA injections.

Dose Gorup	MT10107·100U		BOTOX·50U	
	Volume of Dilution	Volume of Injection	Volume of Dilution	Volume of Injection
2U Group	5.0 mL	0.1 mL	2.5 mL	0.1 mL
5U Group	2.0 mL	0.1 mL	1.0 mL	0.1 mL
10U Group	1.0 mL	0.1 mL	0.5 mL	0.1 mL
20U Group	0.5 mL	0.1 mL	0.25 mL	0.1 mL
30U Group	0.33 mL	0.1 mL	0.17 mL	0.1 mL

All 25 patients were randomly assigned to the five groups, and each group was arranged to have five subjects. After screening tests, physical examination and baseline measurement, the subjects were injected with a randomly assigned dose of MT10107 into the EDB muscle of a randomly chosen side of foot and an equivalent dose of onabotulinumtoxin. For blind injection, all injections were done with 0.1 mL. MT10107·100U and BOTOX·50U were used for comparison into the identical muscle of a contralateral foot. The injections were done in a randomized order. All of the injections for the participants were performed by the same individual who was blind to the kind of injected agents.

The primary outcome variable was the CMAP amplitudes of the EDB muscle, which is a common neurological model to show efficacy of BoNT/A [10]. It is known to be sensitive to present significant changes between treatments in terms of duration and amount of effects [11]. Efficacy was measured by determining the paretic effects on the EDB muscle after the injection by calculating percentage change of the CMAP amplitudes.

To measure the amplitudes of CMAP, a Sierra wave electromyography machine (Cadwell, WA, USA) was used. All responses were recorded with supramaximal electrical stimulation of the peroneal nerve at the ankle. Single electrical stimulation of 0.2 ms was given, and the amplitude was measured in the area of maximum response. For filter setting, low frequency filter was set to 2 Hz and high frequency filter to 10,000 Hz. Surface electrodes positions and the setting for electromyography were maintained constant on every visit. The skin temperatures of the patients were between 32 °C and 34 °C. The same examiner, who was blind to the injection materials, performed all measurements.

For all visits, including the injection day, Day 14, 30, and 90 after the injection, electromyographic measurements were performed on the EDB, AH, and ADQ muscles, compared with the baseline values which were measured before the injection. After the injection, each subject was tested three times, and the paralytic effect was confirmed by the percentage reduction of the CMAP M-wave amplitude compared to the baseline. For electromyography, base-to-peak amplitude of the CMAP M-wave was measured on EDB, AH, and ADQ using surface electrodes. The CMAP M-waves were measured three times, and the two largest values were averaged and utilized.

In addition, antibody tests against BoNT were done before the injection and 90 days after the injection. Immunogenicitytests were performed using serum samples from the subjects to identify the formation of anti-MT10107 antibodies. Mouse protection assays were conducted. The specific neutralizing antibodies to MT10107 are detected by mouse protective bioassay. Mouse protective bioassay is the method to detect the ability of serum to neutralize lethality of the MT10107. Pre-incubated MT10107 with serum samples of subjects are intraperitoneally injected into mouse. Mouse lethality is checked during the test dates (four days post-injection). If three or more mice of four mice lived, then the injected serum would have neutralizing antibody. At visit 1 and visit 6, blood samples were taken for immunogenicity testing per subject. The collected sera of subjects were stored in deep freezers maintained at approximately −70 °C. Before the injection, and at Day 14, 30, and 90, blood chemistry tests were performed including blood WBC, RBC, hemoglobin, platelets, neutrophils, lymphocytes, monocytes, eosinophils, basophils, sodium, chloride, potassium, calcium, BUN, creatinine, uric acid, total bilirubin, ALT, AST, LDH, alkaline phosphatase, total cholesterol, phosphorus, total protein, albumin, and glucose levels. Also, urines were assessed for urine pH, specific gravity, protein, glucose, nitrites, bilirubin, urobilinogen, ketones, occult blood, WBC counts. On each of the four visits, physical examination was done including vital signs, as well as an electrocardiogram.

### 4.3. Efficacy and Safety Parameters

The primary outcome variable was the percentage reduction of the CMAP M-wave amplitude on the bilateral EDB muscles compared to the baseline measurements. To access the local diffusion of the drugs, the CMAP M-wave amplitudes were determined, recording on the adjacent muscles, AH and ADQ, near injected EDB muscles.

The safety was evaluated by physical and neurological examinations including presence of any local side effects of the lower extremities, any abnormal vital signs, laboratory parameters, electrocardiogram, general complications, and the antibody test at every visit; and the patients were interviewed 60 days after the injection.

### 4.4. Statistical Analyses

To access efficacy, the differences between the study and the control groups were determined by the paired *t*-test. Because of a small number of participants in each group (*n* = 5), there were non-normally distributed groups; therefore, either paired *t*-test or Wilcoxon signed-rank test was used due to the distribution. The significance of the percentage reduction of the EDB CMAP M-wave amplitudes were compared to those of the baseline. The percentage reductions among the dose groups, compared with the baseline, were evaluated by ANOVA (Analysis of Variance). For the local diffusion assessments, the CMAP M-wave amplitudes of AH and ADQ, the adjacent muscles, were measured. The significances of the percentage reduction of the amplitudes were compared to the baseline. The differences between the study and the control groups were tested via the paired *t*-test and Wilcoxon’s signed rank test. Differences among the various dose groups for the incidence of local diffusion were tested by Fisher’s exact test. SAS 9.1 (SAS Institude Inc., Cary, NC, USA) was used for all statistical analysis, and *p*-value less than 0.05 was considered statistically significant.

## 5. Conclusions

This study indicates that MT10107 is as effective as onabotulinumtoxinA. Compared to onabotulinumtoxinA, our control agent, there was no significant difference in efficacy and local diffusion.
